# Structures of three ependymin-related proteins suggest their function as a hydrophobic molecule binder

**DOI:** 10.1107/S2052252519007668

**Published:** 2019-06-20

**Authors:** Jeong Kuk Park, Keon Young Kim, Yeo Won Sim, Yong-In Kim, Jin Kyun Kim, Cheol Lee, Jeongran Han, Chae Un Kim, J. Eugene Lee, SangYoun Park

**Affiliations:** a School of Systems Biomedical Science, Soongsil University, Seoul 06978, Republic of Korea; b Center for Bioanalysis, Korea Research Institute of Standards and Science, Daejeon 34113, Republic of Korea; c Department of Physics, Ulsan National Institute of Science and Technology, Ulsan 44919, Republic of Korea; d Department of Biological Sciences, Korea Advanced Institute of Science and Technology, Daejeon 34141, Republic of Korea

**Keywords:** ependymin, mammalian ependymin-related protein, UCC1, protein structure, X-ray structure, structure determination, X-ray crystallography

## Abstract

Ependymin was first discovered as a predominant protein in brain extracellular fluid in fish and was suggested to be involved in functions mostly related to learning and memory. Orthologous proteins to ependymin called ependymin-related proteins (EPDRs) have been found to exist in various tissues from sea urchins to humans, yet their functional role remains to be revealed. In this study, the structures of EPDR1 from frog, mouse and human were determined and analyzed. All of the EPDR1s fold into a dimer using a monomeric subunit that is mostly made up of two stacking antiparallel β-sheets with a curvature on one side, resulting in the formation of a deep hydrophobic pocket. All six of the cysteine residues in the monomeric subunit participate in the formation of three intramolecular disulfide bonds. Other interesting features of EPDR1 include two asparagine residues with glycosylation and a Ca^2+^-binding site. The EPDR1 fold is very similar to the folds of bacterial VioE and LolA/LolB, which also use a similar hydrophobic pocket for their respective functions as a hydrophobic substrate-binding enzyme and a lipoprotein carrier, respectively. A further fatty-acid binding assay using EPDR1 suggests that it indeed binds to fatty acids, presumably *via* this pocket. Additional interactome analysis of EPDR1 showed that EPDR1 interacts with insulin-like growth factor 2 receptor and flotillin proteins, which are known to be involved in protein and vesicle translocation.

## Abstract 2

Ependymin-related proteins fold into a dimer using a subunit formed by stacking antiparallel β-sheets and contain a hydrophobic pocket that is found in the similar folds of bacterial VioE and LolA/LolB. As in VioE and LolA/LolB, this hydrophobic pocket may be used to bind hydrophobic molecules.

## 1. Introduction   

Although ependymin (also known as EPN or EPD) was first discovered in teleost fish (Shashoua, 1976*a* ▸,*b* ▸, 1977 ▸; Benowitz & Shashoua, 1977 ▸), ependymin-related proteins are found widely from sea urchins (Suárez-Castillo *et al.*, 2004 ▸) to humans (Apostolopoulos *et al.*, 2001 ▸; Gregorio-King *et al.*, 2002 ▸; Nimmrich *et al.*, 2001 ▸; Suárez-Castillo & García-Arrarás, 2007 ▸). As the name implies, ependymin was discovered in the ependymal zone of goldfish brain, and its level was found to increase upon a new learning event (Shashoua, 1976*a* ▸,*b* ▸, 1977 ▸; Benowitz & Shashoua, 1977 ▸). Subsequent studies on ependymin showed that it was the most abundant glycoprotein in the brain extracellular fluid (ECF) and cerebrospinal fluid of teleost fish, and that it was involved in various roles related to memory consolidation, neuronal regeneration and brain calcium homeostasis (Shashoua, 1991 ▸; Schmidt, 1995 ▸). Additional studies indicated that fish ependymin has an influence on cold adaptation (Tang *et al.*, 1999 ▸) and aggressiveness (Sneddon *et al.*, 2011 ▸). Although the detailed cellular mechanisms underlying the functions of fish ependymin still remain elusive, the existence of several Ca^2+^-binding sites led to the belief that it is a secreted extracellular matrix protein (Schmidt, 1995 ▸; Ganss & Hoffmann, 1993 ▸, 2009 ▸; Hoffmann & Schwarz, 1996 ▸). Studies using a peptide fragment of fish ependymin suggested that it activates c-Jun N-terminal kinase and the downstream AP-1 transcription factor in murine nerve cells (Shashoua *et al.*, 2001 ▸; Adams *et al.*, 2003 ▸; Kaska, 2003 ▸).

Orthologous proteins to fish ependymin also exist in other vertebrates, including mammals. For instance, proteins named mammalian ependymin-related protein 1 (known as MERP1) exist in both mouse and human. Unlike the brain-specific expression in fish, the orthologues in mouse and human were expressed in various normal tissues as well as in cancerous cell lines (Apostolopoulos *et al.*, 2001 ▸; Gregorio-King *et al.*, 2002 ▸). In another study, the human form showed an increased transcription level in colorectal tumor cells and hence was named UCC1 (upregulated in colorectal cancer gene 1; Nimmrich *et al.*, 2001 ▸). Additionally, the results of mouse phenotyping (International Mouse Phenotyping Consortium; http://www.mousephenotype.org/data/genes/MGI:2145369) indicated that both male and female homozygote MERP1-knockout mice were normal apart from significant decreases in tibia length (*P* = 3.2 × 10^−7^), locomoter activity (hypoactivity; *P* = 3.9 × 10^−7^) and fat mass (*P* = 7.4 × 10^−6^). Hereafter, ependymin and its orthologues will all be referred to as ependymin-related proteins (EPDRs) for simplicity.

In contrast to fish EPDR, which is secreted into the ECF, the cellular fate of human or rodent EPDR1 was assigned as lysosomal localization. Most luminal lysosomal proteins that are folded and processed in endoplasmic reticulum (ER) and the Golgi complex are targeted specifically to the lysosome by mannose 6-phosphate (M6P) tagging and their recognition and sorting by M6P receptors (known as MPRs). Human and rodent EPDR1s were found in multiple proteomic analyses of lysosomal proteins isolated using MPR-immobilized beads (Sleat *et al.*, 1996 ▸, 2005 ▸, 2006 ▸; Kollmann *et al.*, 2005 ▸; Tribl *et al.*, 2005 ▸), and the isolated EPDR1 was shown to contain the M6P modification (Kollmann *et al.*, 2005 ▸; Sleat *et al.*, 2006 ▸; Lübke *et al.*, 2009 ▸). Furthermore, a lipidosis-induced density shift experiment demonstrated quite conclusively that mouse EPDR1 resides within the lysosome (Della Valle *et al.*, 2006 ▸).

The protein sequences of all EPDRs generally contain an ER-targeting signal-peptide sequence at the N-terminus (Müller-Schmid *et al.*, 1992 ▸). The sequences of EPDRs also contain four to six highly conserved cysteine residues that are predicted to form disulfide cross-links (Müller-Schmid *et al.*, 1992 ▸; Apostolopoulos *et al.*, 2001 ▸). Fish EPDR was found to be glycosylated at two asparagine sites (Benowitz & Shashoua, 1977 ▸; Shashoua, 1977 ▸; Königstorfer *et al.*, 1989 ▸), and all known EPDR sequences from other organisms contain at least two predicted N-glycosylation sites that do not necessarily align with the glycosylation sites in fish.

Despite the fact that EPDRs are conserved across species (Suárez-Castillo & García-Arrarás, 2007 ▸), studies providing clues to their function are limited to that from fish, and the detailed mechanism of action of EPDR remains to be revealed. At this point, a homology search using the EPDR protein sequence failed to show any significant similarity to other proteins of known function. Hence, EPDR represents a particularly interesting protein that requires further structural investigation in order to predict its function. In this attempt, frog (*Xenopus tropicalis*) EPDR1, mouse (*Mus musculus*) EPDR1 and human EPDR1, all without the signal-peptide sequence, were recombinantly expressed in insect cells and their structures were determined to 2.0, 2.4 and 2.0 Å resolution, respectively, and analyzed. During the course of writing up this paper, Wei and coworkers reported a 3.0 Å resolution human EPDR1 structure (Wei *et al.*, 2019 ▸). Hence, we refer to their results and also compare their results with ours here.

## 2. Experimental procedures   

### 2.1. Plasmid cloning   

DNAs for human EPDR1 (UniProt ID Q9UM22; residues 38–224), mouse EPDR1 (UniProt ID Q99M71; residues 38–224) and frog EPDR1 (*X. tropicalis*; NCBI Accession No. XP_002939463; residues 38–220), which all exclude the native N-terminal signal-peptide sequences, were gene-synthesized (Bioneer, Daejeon, Republic of Korea) with the addition of N-terminal His_8_ tags and BamHI/NotI restriction-enzyme sites. The genes were also codon-optimized for expression in the *Spodoptera frugiperda* (Sf9) insect-cell line. The synthesized DNAs were subcloned into pAcGP67A vector (BD Biosciences, Franklin Lakes, New Jersey, USA) for secreted expression.

### 2.2. Protein expression   

The conventional method of insect-cell expression using the Sf9 insect-cell line and baculovirus was used to obtain the three ependymin-related (EPDR1) proteins. The insect-cell culture was performed in a 27°C incubator or a shaker using Corning Insectagro medium (Thermo Fisher Scientific, Waltham, Massaschusetts, USA) supplemented with 1× Gibco Antibiotic Antimycotic (Thermo Fisher Scientific). Generations of baculovirus encoding the three EPDR1s were performed by co-transfecting each subcloned plasmid and the baculovirus DNA (BestBac Linearized Baculovirus DNA, Expression Systems, Davis, California, USA) into Sf9 cells according to the manufacturer’s instructions. Further virus amplifications through multistep infections were subsequently performed until fourth-passage virus stocks were obtained. Approximately 1 l of Sf9 cells (2 × 10^6^ cells ml^−1^) were infected using the final virus stocks, and the cells were harvested after two days when the maximum amounts of proteins were found to be secreted into the supernatant. Detailed methods for the expression of frog EPDR1 have been reported elsewhere (Park *et al.*, 2018 ▸).

### 2.3. Protein purification   

The three EPDR1s were purified using nickel-affinity chromatography *via* the N-terminal His_8_ tags designed within the recombinant proteins, followed by size-exclusion chromatography (Park *et al.*, 2018 ▸). Firstly, stock solutions of 1 *M* Tris pH 7.5 and 5 *M* NaCl were used to bring the supernatants to 50 m*M* Tris pH 7.5 and 200 m*M* NaCl, and the pH was adjusted to pH 7. For about 1 l of harvested supernatant, 20 ml Ni–NTA (nitrilotriacetic acid) agarose resin (Qiagen, Hilden, Germany) was used for protein binding. The protein-bound resin was further washed with 100 ml wash buffer (20 m*M* imidazole, 25 m*M* Tris pH 7.5, 500 m*M* NaCl) and the protein was eluted using elution buffer (200 m*M* imidazole, 25 m*M* Tris pH 7.5, 500 m*M* NaCl). Proteins in the collected fraction were checked using SDS–PAGE and concentrated using an Amicon Ultra-15 centrifugal filter (Millipore; Merck, Kenilworth, New Jersey, USA) to 10 ml, which was optimal for loading onto a size-exclusion column (Superdex 200 HR26/60; GE Healthcare, Little Chalfont, England) that had been pre-equilibrated with gel-filtration buffer (GFB; 50 m*M* Tris pH 7.5, 150 m*M* NaCl). The elution chromatograms of the three EPDR1s all showed single-peak profiles (Supplementary Fig. S1). The elution fractions were concentrated to a protein concentration of 5–10 mg ml^−1^. The absorptivity coefficients (∊) of the three EPDR1s at λ = 280 nm were calculated from the numbers of tyrosine and tryptophan residues in the proteins: human, 1.8 mg^−1^ ml cm^−1^; mouse, 1.8 mg^−1^ ml cm^−1^; frog, 1.6 mg^−1^ ml cm^−1^. The overall yields of the purified EPDR1 proteins were marginal: 0.5–1.5 mg per litre of insect-cell culture. The final proteins were checked again for homogeneity on SDS–PAGE (Supplementary Fig. S1). Interestingly, the size of human EPDR1 on SDS–PAGE was smaller than those of mouse and frog EPDR1 (Supplementary Fig. S1). The three proteins were aliquoted, flash-frozen in liquid nitrogen and stored in a −80°C deep-freeze for crystallization and further assays.

### 2.4. Crystallization   

The three EPDR1s were screened for crystallization using commercial screening solutions (Hampton Research, Aliso Viejo, California, USA) by the hanging-drop method at 22°C. Optimized single crystals of human EPDR1 appeared in well reservoirs consisting of 15–20%(*w*/*v*) PEG 3350, 0.1 *M* citric acid pH 4.5 or of 15–20% PEG 3350, 0.2 *M* NaCl, 0.1 *M* bis-Tris pH 5.5. Optimized single crystals of mouse EPDR1 appeared in a well reservoir consisting of 0.5–1.0 *M* lithium sulfate, 0.5–1.0 *M* ammonium sulfate, 0.1 *M* sodium citrate pH 5.6. Optimized single crystals of frog EPDR1 appeared in a well reservoir consisting of 15–20% PEG 8000, 0.2 *M* calcium acetate, 0.1 *M* sodium cacodylate pH 6.5. All three crystal forms appeared in 1–3 days in hanging drops at 22°C. The crystals were transferred into cryoprotectant solutions, which were made by adding glycerol to the reservoir solution to a final concentration of 20%, and were flash-cooled in liquid nitrogen for storage and transport to a high-pressure or X-ray synchrotron facility.

### 2.5. Xenon pressurization of frog EPDR1 crystals for phase determination   

A single crystal of frog EPDR1 pressurized with xenon gas was used for phase determination. Xenon pressurization of the crystals was carried out using a high-pressure cryocooler by modifying the high-pressure cryocooling method of Kim *et al.* (2005 ▸). Crystals mounted in cryoloops were placed in the bottom part of the high-pressure tubing blocked with an end cap. The upper end of the tubing was then connected to the xenon-gas cylinder. After the high-pressure tubing had been firmly connected to the high-pressure cryocooler, 1 MPa xenon pressure was applied to the crystal and equilibrated for 5 min at room temperature. While maintaining the pressure, the liquid-nitrogen bath was quickly lifted up to three-quarters of the height of the tubing to cryocool the crystal. The crystal was cooled for about 2 min in the tubing, the remaining xenon pressure was released and the crystal was transferred into cryocaps under liquid nitrogen for transport to the synchrotron.

### 2.6. X-ray data collection and structure determination   

X-ray diffraction data were collected at 100 K using CCD detectors (ADSC Quantum 270 and 315) on beamlines 7A and 5C at Pohang Light Source (PLS), Pohang, Republic of Korea (Table 1 ▸). All data were processed and scaled using *HKL*-2000 (Otwinowski & Minor, 1997 ▸). The phases of the structure factors for the frog EPDR1 crystal were determined using single-wavelength anomalous diffraction (SAD) from a single Xe-derivatized crystal with data collected at λ = 1.54 Å. Although *f*′′ at λ = 1.54 Å is only half the maximum at the Xe absorption edge, the anomalously scaled data had sufficient signal for phasing. The anomalously scaled data at 2.9 Å resolution were analyzed in *PHENIX* (Adams *et al.*, 2010 ▸), where five Xe sites were located for experimental phasing. Automatic experimental phasing followed by density modification using *PHENIX* led to an interpretable electron-density map showing multiple β-sheeted folding of the protein, and automatic model building within *PHENIX* generated an initial model. The *PHENIX*-generated model was then subjected to an automated *ARP*/*wARP* (Langer *et al.*, 2008 ▸) building cycle via a web service using higher resolution (2.0 Å) diffraction data from the frog EPDR1 crystal, in which more complete model building was performed. Manual model inspection and corrections of the structure using the electron-density map were performed in *Coot* (Emsley & Cowtan, 2004 ▸). Only one molecule of EPDR1 was found in the asymmetric unit of the frog EPDR1 crystal. The structures of human and mouse EPDR1 were determined using the frog EPDR1 model via molecular replacement performed with *Phaser* (McCoy *et al.*, 2007 ▸). Four molecules of EPDR1 were found in the asymmetric units of both the mouse and human EPDR1 crystals. Manual model corrections and building into the structure in these cases were also performed in *Coot*. The final refinements of the three structures were performed using *REFMAC*5 (Murshudov *et al.*, 2011 ▸) with no data cutoff. Structures were analyzed in *Coot* and *PyMOL* (Schrödinger, New York, USA) and structural figures were rendered in *PyMOL*. The topology diagram was generated using the *Pro-origami* web server (Stivala *et al.*, 2011 ▸) and was modified for our figure. Simulated-annealing OMIT maps were created in *PHENIX*.

### 2.7. Enzyme assay   

Lipase and phospholipase assays using the purified human EPDR1 were performed according to the manufacturers’ protocols. EPDR1 did not show any activities using the Lipase Activity Assay Kit (catalog No. MAK046; Sigma–Aldrich, St Louis, Missouri, USA), the Phospholipase D Activity Assay Kit (catalog No. MAK137; Sigma–Aldrich) or the EnzChek Direct Phospholipase C Assay Kit (catalog No. E10215; Thermo Fisher Scientific, Waltham, Massachusetts, USA).

### 2.8. Fatty-acid-binding assay   

A fluorescent probe (1-anilinonaphthalene-8-sulfonic acid; 1,8-ANS) displacement assay of human EPDR1 was performed using three saturated fatty acids [caproic acid (C6), lauric acid (C12) and stearic acid (C18)]. All chemicals were purchased from Sigma–Aldrich (St Louis, Missouri, USA). The 1,8-ANS binding and displacement assays were based on methods described in previous studies of fatty-acid-binding proteins (Kane & Bernlohr, 1996 ▸; Shimamoto *et al.*, 2014 ▸). The binding of 1,8-ANS to human EPDR1 was first measured by the increase in fluorescence (excitation at 355 nm, emission at 460 nm) on the addition of 1,8-ANS to a fixed 1 µ*M* concentration of human EPDR1 in GFB. Subsequent displacements of 1,8-ANS inferred by the decrease in fluorescence were measured by adding the three fatty acids to mixtures of 1 µ*M* human EPDR1 and 50 µ*M* 1,8-ANS in GFB. Increasing concentrations of caproic acid or lauric acid were added to mixtures of human EPDR1 and 1,8-ANS, all in a final concentration of 0.5% ethanol in GFB. For stearic acid, with a lower water solubility, the experiment was performed in a final concentration of 2% ethanol in GFB. The experiments were performed in 96-well plates with 100 µl final volume and were kept at 25°C in the dark for 3 min before measuring the fluorescence on a multiple plate reader (Wallac Victor 3, Perkin Elmer, Waltham, Massachusetts, USA). The dissociation constant (*K*_d_) and IC_50_ (concentration at 50% inhibition) values were calculated using a web-based IC_50_ toolkit (http://ic50.tk).

### 2.9. Identification of the EPDR1 interactome   

To analyse the interactome of EPDR1, transient expression of EPDR1 was induced using a pcDNA5/FRT vector (Thermo Fisher Scientific, Waltham, Massachusetts, USA) encoding full-length human EPDR1 (1–224) including the N-terminal signal peptide. The human EPDR1 gene was cloned into the vector using NheI and BamHI sites. The PCR reaction was carried out using a purchased human EPDR1 gene (MGC Human EPDR1, Clone ID 3461888; Dharmacon, Lafayette, Colorado, USA). A linker (GGGGS) and a FLAG-tag (DYKDDDDK) were added at the C-terminus of the EPDR1 gene (EPDR1^FLAG^) by inserting DNA sequences encoding the linker and the tag into the 3′ PCR primer.

U-87MG cells were plated onto a 150 mm dish in DMEM. 30 µg of an empty pcDNA5/FRT vector or the vector containing the EPDR1^FLAG^ sequence were transfected into the U-87MG cells using Lipofectamine 2000 (Life Technologies, Baltimore, Maryland, USA) and Opti-MEM (Life Technologies) according to the manufacturer’s instructions. After 48 h of incubation, the control medium or the medium containing the secreted EPDR1^FLAG^ was concentrated using 10 kDa molecular-weight cutoff Amicon Ultra-15 filter units (Millipore, Cork, Ireland).

1 mg of protein from the medium was incubated with anti-FLAG M2 agarose beads (Sigma–Aldrich, St Louis, Missouri, USA) at 4°C for 2 h. U-87MG cell lysates were prepared using cells not subjected to transient expression of EPDR1^FLAG^. The U-87MG cells were harvested and lysed in lysis buffer consisting of 50 m*M* HEPES pH 7.5, 150 m*M* NaCl, 70 m*M* potassium acetate, 5 m*M* magnesium acetate, 1% *n*-dodecyl β-d-maltoside (Thermo Fisher Scientific, Rockford, Illinois, USA) and protease-inhibitor cocktail (Roche, Mannheim, Germany) at 4°C for 30 min. The cell lysates were centrifuged at 16 000*g* for 20 min. 5 mg protein from the supernatant was incubated at 4°C for 1 h with M2 agarose beads pre-bound with the medium prepared from the U-87MG cell culture transiently expressing EPDR1^FLAG^. The beads were washed five times with the lysis buffer and then twice with 100 m*M* Tris–HCl pH 8.5. The FLAG immunoprecipitates were prepared for mass spectrometry as described previously (In *et al.*, 2019 ▸). Briefly, the bound proteins were eluted from the beads using 10 *M* urea, reduced with tris(2-carboxyethyl)­phosphine and alkylated with 2-chloroacetamide. The proteins were digested by Lys-C endoprotease (Wako, Osaka, Japan) at 37°C for 6 h before being further digested by trypsin (Promega, Madison, Wisconsin) at 37°C for 13 h. The digested peptides were desalted on reverse-phased C18 Stage Tips (Rappsilber *et al.*, 2007 ▸). The resulting eluates were dried in a vacuum concentrator and resuspended in 0.1% formic acid.

Liquid chromatography–mass spectrometry analyses were performed with an EASY-nLC 1000 coupled to a Q-Exactive Orbitrap mass spectrometer (Thermo Scientific, San Jose, California, USA) equipped with a custom electrospray ionization source. Digested peptides were separated on a 150 mm reversed-phase analytical column (75 µm internal diameter) packed with C18 AQ resin (3 µm, 10 nm; Bonna-Agela Technologies, Wilmington, Delaware, USA). The separation took 120 min using a nonlinear gradient of 4.5–85.5% aceto­nitrile at a flow rate of 350 nl min^−1^. The mass spectrometer was automatically switched between full-scan MS and tandem MS acquisition in a data-dependent mode. Full-scan survey mass spectra were collected (*m*/*z* 300–1800) in an Orbitrap utilizing an automated gain-control target of three million ions with a resolution of 70 000. Tandem mass spectra were acquired using an automated gain-control target of a half a million ions with a resolution of 17 500. The top 12 most intense ions were isolated for fragmentation by higher-energy collisional dissociation. All single-charged and charge-unassigned precursor ions were discarded.

MS peaks were generated from raw MS files using *MaxQuant* (v.1.6.0.1). The *Andromeda* peptide-search engine in *MaxQuant* was used to match the MS peaks against a concatenated UniProt human database (October 2017 release) and a decoy database constructed with modified reversing of protein sequences as described previously (Cox & Mann, 2008 ▸). The search parameters were trypsin digestion, fixed carboxyamidomethyl modifications of cysteine, a maximum of two missed cleavages, variable oxidation of methionine, variable acetylation of protein N-termini, variable deamidation of asparagine and glutamine, and variable carbamylation of peptide N-termini. The mass tolerances were 4.5 p.p.m. and 20 p.p.m. for precursor and fragment ions, respectively. Protein inference and quantitation were performed using *MaxQuant* with a 1% false discovery rate (FDR) threshold for both peptides and proteins. Abundances of the identified proteins were inferred from the MaxLFQ intensity (Cox *et al.*, 2014 ▸). Statistics and visualization were performed using *Perseus* (v.1.6.0.2; Tyanova *et al.*, 2016 ▸). The statistical significance of protein abundance difference was determined using Student’s *t*-test (FDR < 0.05, *S*_0_ = 1).

## 3. Results and discussion   

### 3.1. Overall structure   

Three homologous EPDR1s from human, mouse and frog were expressed in insect cells, purified and crystallized (Supplementary Fig. S1). The structures of the three EPDR1s were determined to resolutions of 2.0, 2.4 and 2.0 Å, respectively (Table 1 ▸). The frog EPDR1 structure was determined first by direct phasing using single-wavelength anomalous diffraction (SAD) from a xenon-derivatized crystal, and the structure was subsequently used as a search model to determine the mouse and human EPDR1 structures by molecular replacement. A total of four monomeric subunits of EPDR1 were located in the asymmetric units of the human and mouse EPDR1 crystals. The refined structures showed average C^α^ r.m.s.d.s of 0.5 Å among the four human EPDR1 molecules and 1.0 Å among the four mouse EPDR1 molecules. Only one molecule of frog EPDR1 was found in the asymmetric unit of the crystal of frog EPDR1. An alignment of the three EPDR1 sequences based on the determined structures with identity percentages is shown in Supplementary Fig. S2.

**Figure 1:**
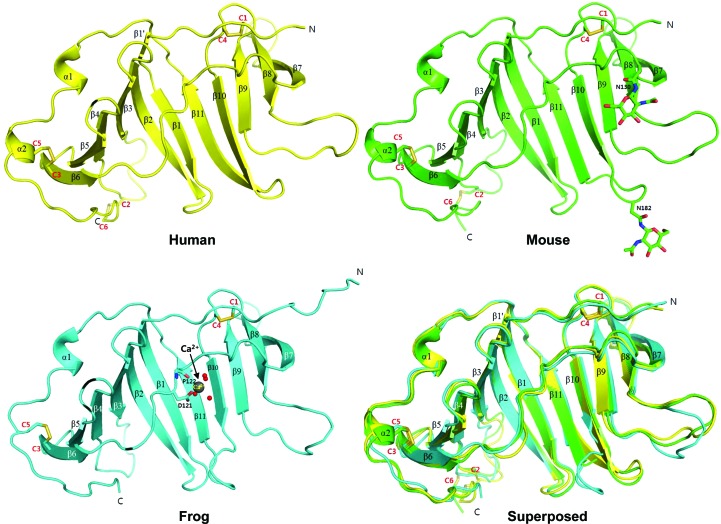
The overall structures of human, mouse and frog EPDR1. A ribbon diagram of human EPDR1 is shown (in yellow) with secondary-structure elements indicated. The locations of cysteines (C1–C6) and the disulfide bonds are also shown. A ribbon diagram of mouse EPDR1 is shown (in green) with two asparagine residues and their NAG glycosylation shown as stick models. Although other types of glycosylation were observed at Asn182, only NAG is shown for clarity (see Fig. 3 ▸ for more on glycosylation). A ribbon diagram of frog EPDR1 is shown (in blue) with Ca^2+^ ions (shown as spheres), four Ca^2+^-binding waters (shown as spheres) and direct Ca^2+^-interacting residues (shown in stick representation). The details of the interaction network stabilizing the bound Ca^2+^ ions in frog EPDR1 are shown in Fig. 3 ▸(*c*). Note that the same colors will be used throughout the figures. The superposed structures of the three EPDR1s are also shown. The superimposed EPDR1 structures show C^α^ r.m.s.d.s of 0.8 Å (human versus mouse), 1.1 Å (human versus frog) and 1.1 Å (mouse versus frog).

All three structures show identical folds comprised of two stacked β-sheet layers created by 11 β-strands arranged in an antiparallel fashion (β6–β5–β4–β3–β2–β1–β11–β10–β9–β8–β7; Fig. 1 ▸). The first layer of the β-sheet is created by β1–β6 (first layer; β6–β5–β4–β3–β2–β1). The remaining β-strands β7–β11 along with β1 and β2 form the second layer (β2–β1–β11–β10–β9–β8–β7; Supplementary Fig. S3). The two β-sheet layers extend side-by-side with partially open surfaces, and the two long and bent β2 and β1 strands in the middle of the fold create the curvature between the two layers (Fig. 1 ▸). The two β-sheet layers are connected by a 14-residue loop (the β6–β7 loop) that crosses over to link β6 and β7 at the opposite sides. While the first β-sheet layer provides a concave surface at the center of the protein, the second β-sheet layer mediates the dimeric interface (as discussed later). Two tandem α-helices (α1 and α2), which are located at the C-terminal end following β11, surround the first β-sheet layer. All six cysteine residues in the EPDR1 sequences participate in forming three disulfide bonds (Fig. 1 ▸ and Supplementary Fig. S2): (i) Cys42 (human sequence, labeled C1) at the N-terminal end and Cys172 (human sequence, labeled C4) in the β9–β10 loop, (ii) Cys88 (human sequence, labeled C2) in the β3–β4 loop and Cys222 (human sequence, labeled C6) at the C-terminal end and (iii) Cys113 (human sequence, labeled C3) in β6 and Cys210 (human sequence, labeled C5) in α2.

Only small differences in the overall structure are observed among the EPDR1s from human, mouse and frog (Fig. 1 ▸). For instance, the long bent β1 of the mouse and frog EPDR1s, which is involved in the formation of the first and second β-sheet layers along with β2, is divided into two β-strands in human EPDR1 (labeled β1′ and β1 in Fig. 1 ▸). Also, the β8–β9 and β10–β11 loops in frog EPDR1 diverge significantly in comparison to those from human and mouse. In particular, the displacement of the β10–β11 loop towards β1 in frog EPDR1 confers an altered conformation of the shortened β10 and β11. Lastly, α2 at the C-terminus of the human and mouse EPDR1s extends into a loop in frog EPDR1.

### 3.2. Dimerization of EPDR1   

**Figure 2:**
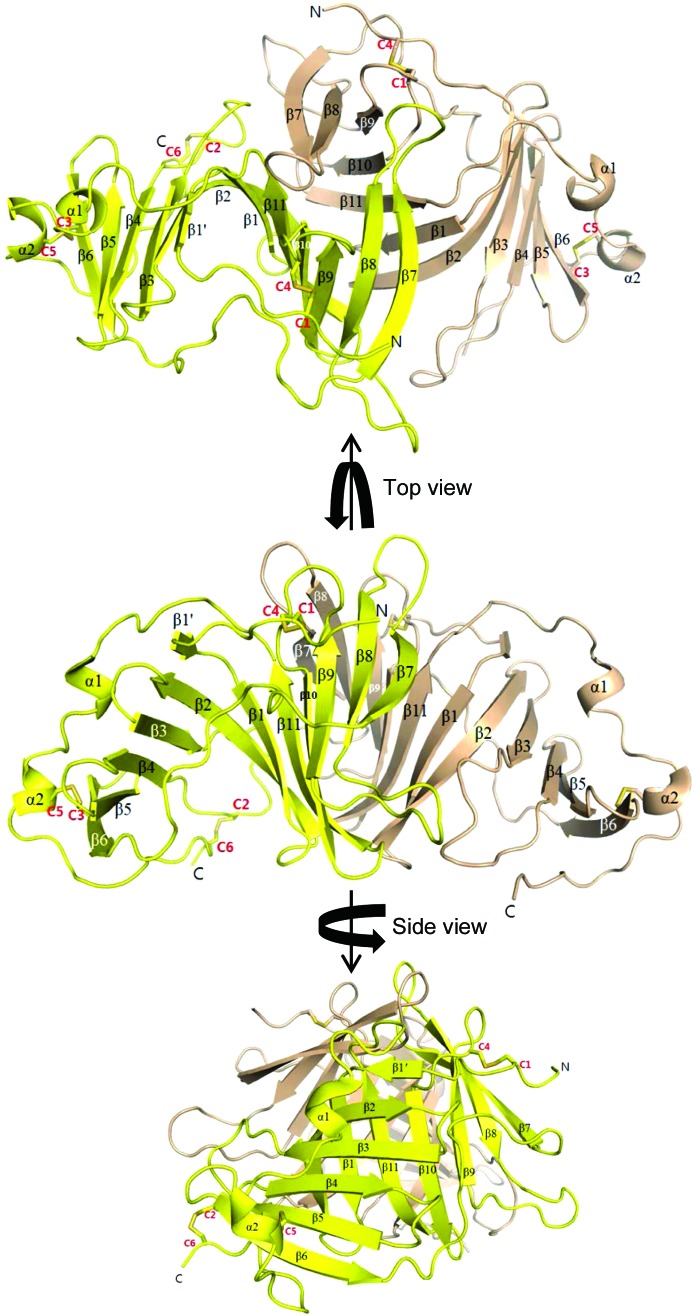
The overall dimeric structure of human EPDR1. Ribbon diagrams of human EPDR1 as dimers are shown as three different views. Secondary-structure elements are labeled and the locations of cysteine (C1–C6)-mediated disulfide bonds are also shown.

In the asymmetric unit of the human EPDR1 crystal, four monomeric subunits of EPDR1 were found, which were associated with other subunits by two perpendicular twofold axes [Supplementary Fig. S4(*a*)]. Buried surface areas (BSAs) of ∼1900 and ∼200 Å^2^ (both per subunit from *PISA* analysis; Krissinel & Henrick, 2007 ▸) were created between the subunits. Judging from the sizes of these areas, the smaller interface burying ∼200 Å^2^ is only a lattice contact, while the dimeric surface burying ∼1900 Å^2^ is likely to be a crucial interaction mode with physiological relevance. In this mode of inter­action, the second of the two β-sheet layers (β2–β1–β11–β10–β9–β8–β7) mediates the dimeric interface (Fig. 2 ▸). This same dimeric interface was conserved in the interaction between the two dimers found in the asymmetric unit of the mouse EPDR1 crystal [Supplementary Fig. S4(*b*)]. BSAs of ∼2300 Å^2^ (per subunit) were calculated for these dimeric interfaces. All other contacts bury a BSA of less than 500 Å^2^, with no conservation in other crystal forms. In the frog EPDR1 crystal with only one monomeric subunit in the asymmetric unit, the same dimeric interface was formed between the crystallographic symmetry mates [BSA of ∼2300 Å^2^ per subunit; Supplementary Fig. S4(*c*)]. The interfacial conservation in all three crystal forms of EPDR1 supports the association of EPDR1 subunits into a dimer using the second β-sheet layer.

### 3.3. Glycosylation and Ca^2+^-binding site   

**Figure 3:**
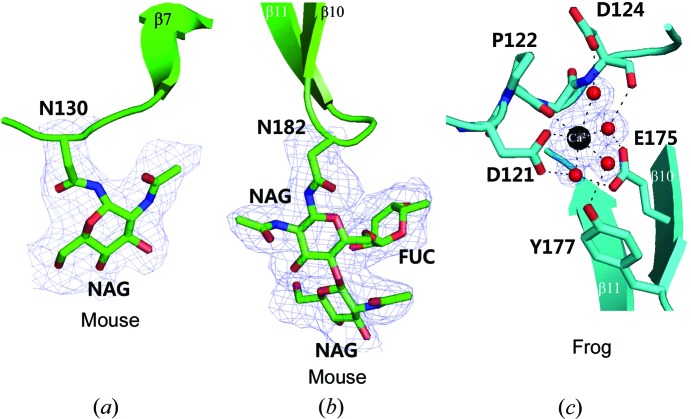
Views of the asparagine residues with glycosylations (in mouse EPDR1) (*a*, *b*) and the Ca^2+^-binding site (in frog EPDR1) (*c*) with experimental electron densities. (*a*) In mouse EPDR1, Asn130 is clearly seen to have a NAG modification. A stimulated-annealing OMIT map of *F*_o_ − *F*_c_ difference density was contoured at 1.1σ. (*b*) Also in mouse EPDR1, Asn182 is clearly seen to have a NAG–FUC–NAG modification. A stimulated-annealing OMIT map of *F*_o_ − *F*_c_ difference density was contoured at 1.1σ. (*c*) In frog EPDR1, Ca^2+^ was found to be octahedrally coordinated by four water molecules and other nearby atoms of Asp121 and Pro122. The water molecules coordinated to Ca^2+^ are further stabilized by tight hydrogen-bonding interactions with the nearby residues Asp124, Glu175 and Tyr177. A stimulated-annealing OMIT map of *F*_o_ − *F*_c_ difference density was contoured at 3.0σ.

Fish EPDR showed glycosylation at two asparagine sites (Benowitz & Shashoua, 1977 ▸; Shashoua, 1977 ▸; Königstorfer *et al.*, 1989 ▸), and the human, mouse and frog EPDR1s had two predicted N-glycosylation sites that were all conserved (Supplementary Fig. S2). Although glycosylation at these Asn residues in human and frog EPDR1 was only slightly perceivable and was too disordered to be modeled accurately as sugars, the glycosylation at the two Asn sites (Asn130 and Asn182) was well ordered in the case of mouse EPDR1 [Figs. 3 ▸(*a*) and 3 ▸(*b*)]. While the sugar modification at Asn130 could be modeled with only one *N*-acetylglucosamine (NAG), that at Asn182 was sufficiently ordered to be modeled with two NAGs (β-1,4 glycosidic bond) and one fucose attached to NAG (α-1,4 glycosidic bond). Such sugar modification is as expected for the initial glycosylation pattern in proteins expressed in insect cells.

Also, strong electron density for a metal was observed near Asp121 only in frog EPDR1 [Fig. 3 ▸(*c*)]. Although we do not have direct evidence, we have modeled this metal as a Ca^2+^ ion, which is likely to originate from the 0.2 *M* calcium acetate condition that was only present in the crystallization reservoir used to obtain the frog EPDR1 crystal. A tight water and amino-acid network was observed surrounding the Ca^2+^ ion. The Ca^2+^ ion is octahedrally coordinated by four water molecules, the side-chain O atom of Asp121 and the main-chain O atom of Pro122. The four water molecules coordinated to the Ca^2+^ ion are further stabilized and arranged in place by hydrogen-bonding interactions with the nearby residues Asp124, Glu175 and Tyr177 [Fig. 3 ▸(*c*)]. Interestingly, although Asp121, Pro122, Asp124 and Glu175 are conserved throughout the frog, mouse and human EPDR1s, a phenyl­alanine substitutes for Tyr177 of frog EPDR1 in both mouse and human EPDR1s (Supplementary Fig. S2). Since the hydroxyl group of Tyr177 stabilizes one of the water molecules that bind to the Ca^2+^ ion, it is expected that the mouse and human EPDR1s would be likely to have weaker binding to the metal. Future studies on this metal-binding site may provide insights into the role of the metal in the function of EPDR1.

### 3.4. Structure-similarity search   

The structure of human EPDR1 was subjected to a fold-similarity search using the *DALI* server (Holm & Sander, 1995 ▸), and the top two highest scoring structures were found to be the bacterial VioE (r.m.s.d. = 3.7 Å; *Z*-score = 14.6) and the bacterial LolA (r.m.s.d. = 4.0 Å; Z-score = 12.3). Interestingly, although the sequences of these proteins show low levels of similarity (sequence identity <10%) to EPDR1 (Supplementary Fig. S5), the topologies of the two β-sheet layered structures formed by 11 antiparallel β-strands are strikingly identical except for some differences in the lengths of the β-strands and some insertions of α-helices (Supplementary Figs. S6 and S7).

The highest scoring VioE is one of the multiple enzymes that mediate the biosynthesis of violacein, which is a purple pigment, in *Chromobacterium violaceum*. Although VioE associates into a dimer in the crystal, the dimeric interaction mode differed from that of EPDR1 (Supplementary Fig. S6; Hirano *et al.*, 2008 ▸; Ryan *et al.*, 2008 ▸). The next most similar, LolA, is a lipoprotein localization factor that is found in the periplasm of Gram-negative bacteria. LolA shuttles lipo­proteins released from the ABC transporter LolCDE to the outer-membrane anchored LolB. It is interesting to note that despite having only ∼8% sequence identity, LolA and LolB also have identical folds (Takeda *et al.*, 2003 ▸). Unlike EPDR1 or VioE, LolA (and LolB) is monomeric in the crystal (Supplementary Fig. S7). The *DALI* search results showed no structurally similar proteins to EPDR1 that are of eukaryotic origin. Hence, EPDR1 represents a protein in human and other eukaryotic organisms that uses a fold previously only known in bacteria.

### 3.5. Hydrophobic pocket   

**Figure 4:**
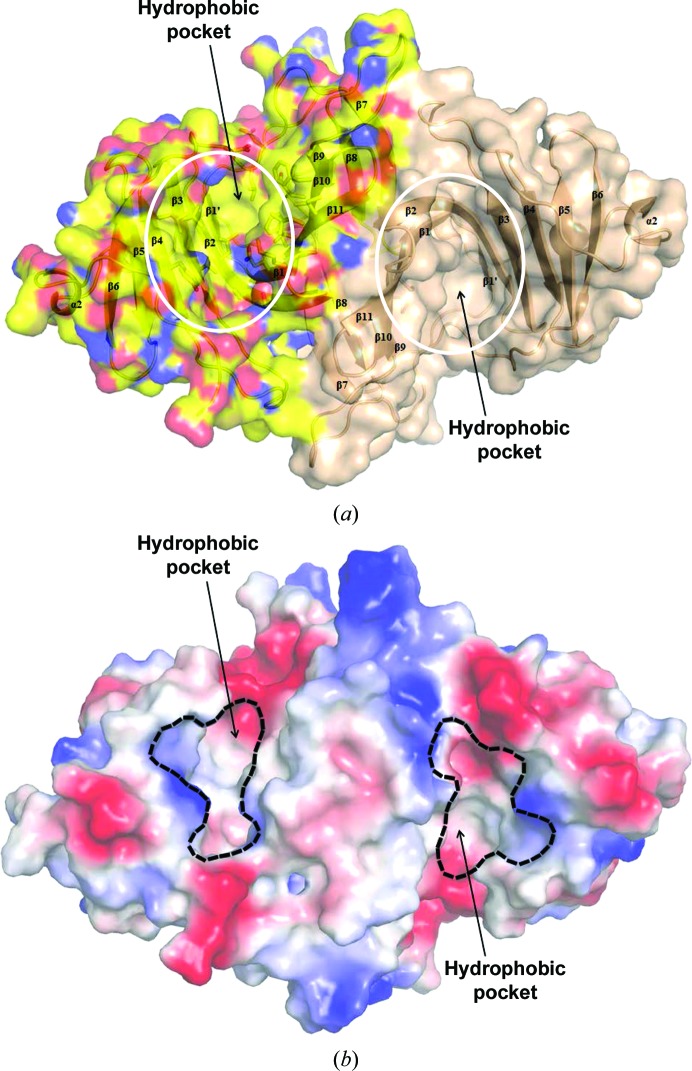
Surface colored by atom type (*a*) and charge-smoothened vacuum contact electrostatic surface (*b*) of human EPDR1. (*a*) The surface rendering (N atoms in blue, O atoms in red and C atoms in yellow) of dimeric human EPDR1 shows a deep hydrophobic cleft. The approximate regions of the hydrophobic pockets are circled in white. (*b*) The surface of negative and positive electrostatic potential patches generated using a charge-smoothened contact potential in *PyMOL* more clearly illustrates the hydrophobic pocket located within each of the monomeric subunits in the EPDR1 dimer. [Note that the negative (red) and positive (blue) charges scaled in *K*_B_ *T*/e_c_ units at pH 7 (*K*_B_, Boltzmann constant; e_c_, charge on the electron) are only qualitatively useful]. The boundaries of the hydrophobic pocket entrances are indicated in black.

When the surface of EPDR1 was analyzed, a deep pocket with a groove volume of ∼6000 Å^3^ (from cleft analysis using *PDBsum*; Laskowski *et al.*, 1997 ▸) was observed (Fig. 4 ▸). This cavity was located on the concave surface of the first β-sheet layer (β6–β5–β4–β3–β2–β1) and was made up of mostly hydrophobic residues. A similar hydrophobic cavity made from the first β-sheet layer also exists in VioE (Supplementary Fig. S6; Hirano *et al.*, 2008 ▸; Ryan *et al.*, 2008 ▸) and LolA/LolB (Supplementary Fig. S7; Takeda *et al.*, 2003 ▸) and is regarded as the pocket necessary for their function. For the enzyme VioE (Hirano *et al.*, 2008 ▸; Ryan *et al.*, 2008 ▸), this pocket was proposed to be the active site for binding and catalyzing the chemical conversion of an as yet unidentified hydrophobic substrate during the final biosynthesis of violacein. For LolA and LolB, which function as lipoprotein carriers, the same pocket was proposed to be the binding site for the acyl chain in the bacterial lipoprotein.

In the structure of LolB (Takeda *et al.*, 2003), continuous electron density was observed in this hydrophobic pocket and was modeled as polyethylene glycol 2000 monomethyl ether (PEG MME 2000), which is likely to originate from the crystallization condition. Also, in a recent structure of human EPDR1 (Wei *et al.*, 2019 ▸) that preceded our study, a ligand was observed in this cavity and was also modeled as an extended PEG chain from the crystallization condition. It is interesting to note that we do not see any electron density for any ligand in the same region despite the fact that PEGs were used to crystallize the human and frog EPDR1s. For the enzyme VioE, a similar region in the pocket contained a PEG molecule that was also likely to arise from the crystallization condition (Supplementary Fig. S6; Ryan *et al.*, 2008 ▸). Several residues near the bound PEG in VioE have been assigned as being critical for its enzyme activity (Hirano *et al.*, 2008 ▸; Ryan *et al.*, 2008 ▸), but none were conserved in EPDR1 (Supplementary Fig. S6).

### 3.6. Functional prediction   

The similarity of the structure of EPDR1 to those of VioE, LolA and LolB, especially in the hydrophobic pocket created by using a single β-sheet layer, which is important for the functions of the lipoprotein carrier proteins (LolA/LolB) and the hydrophobic substrate-binding enzyme (VioE), led us to propose that the eukaryotic EPDR1 may also bind to an as yet uncharacterized hydrophobic molecule for its function. From the clues of a Ca^2+^-binding site found at the mouth of the hydrophobic pocket in frog EPDR1 and of EPDR1 localizing in the lysosome (Della Valle *et al.*, 2006 ▸), we tested EPDR1 for acidic Ca^2+^-dependent lipase and phospholipase activities. However, none were detected.

**Figure 5:**
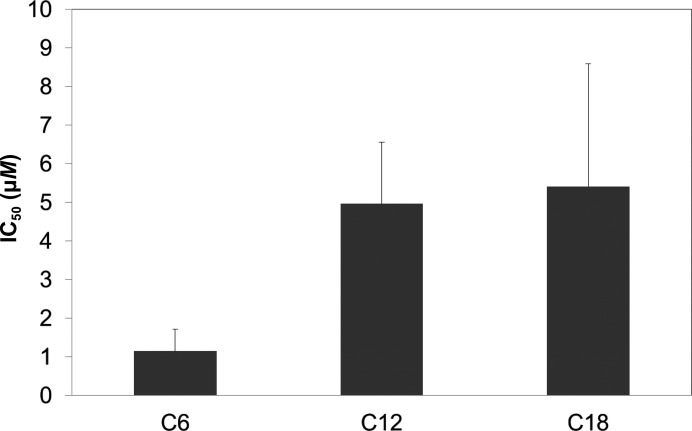
Fatty-acid binding measured by the displacement of a fluorescent probe (1,8-ANS) bound to human EPDR1. Fatty-acid binding to human EPDR1 was inferred by measuring the three fatty-acid (C6, caproic acid; C12, lauric acid; C18, stearic acid) concentrations necessary to replace 50% of 1,8-ANS (IC_50_). Note that the C6 and C12 displacement studies were performed in 0.5% ethanol buffer and that the C18 displacement study was performed in 2% ethanol buffer owing to the limited solubility of C18 in water.

However, when increasing concentrations of a fluorescent 1-anilino­naphthalene-8-sulfonic acid (1,8-ANS) probe were incubated with human EPDR1, the intensity of 1,8-ANS fluorescence increased, suggesting that 1,8-ANS bound to EPDR1 with a *K*_d_ of ∼14 µ*M* (Supplementary Fig. S8). Because the displacement of 1,8-ANS by fatty acids is often used to assess the binding of fatty acids (Kane & Bernlohr, 1996 ▸; Shimamoto *et al.*, 2014 ▸) to fatty-acid-binding proteins, a similar approach was made using human EPDR1 and the fatty acids caproic acid (C6), lauric acid (C12) and stearic acid (C18). The displacement assays suggested that smaller fatty acids more preferably interact with human EPDR1 (Fig. 5 ▸). Because fatty-acid-binding proteins generally interact with fatty acids with a higher binding affinity (*K*_d_ < 5 µ*M*) towards fatty acids with larger sizes (Kane & Bernlohr, 1996 ▸; Shimamoto *et al.*, 2014 ▸), EPDR1 does not seem to be a specific binder of fatty acids but rather a general binder of hydrophobic molecules, and is perhaps important in sequestering digested lipids in lysosomes. In this regard, it remains to be seen whether EPDR1 functions as a hydrophobic molecule-sequestering protein. Also, future structural analysis of EPDR1 in the presence of a lipid may reveal in detail whether the Ca^2+^ ion is directly involved in binding to a lipid.

It is interesting to note that in the recent study by Wei and coworkers human EPDR1 was shown to interact with liposomes containing anionic negatively charged lipids such as bis(monoacylglycero)phosphate (BMP) or ganglioside GM1 at acidic pH (Wei *et al.*, 2019 ▸). In the same study, human EPDR1 was shown to stimulate the activity of neuraminidase-3 while inhibiting neuraminidase-4. From these results, the authors suggested that EPDR1 may function as a lipid transporter or a lysosomal activator protein.

### 3.7. Interactome of EPDR1   

**Figure 6:**
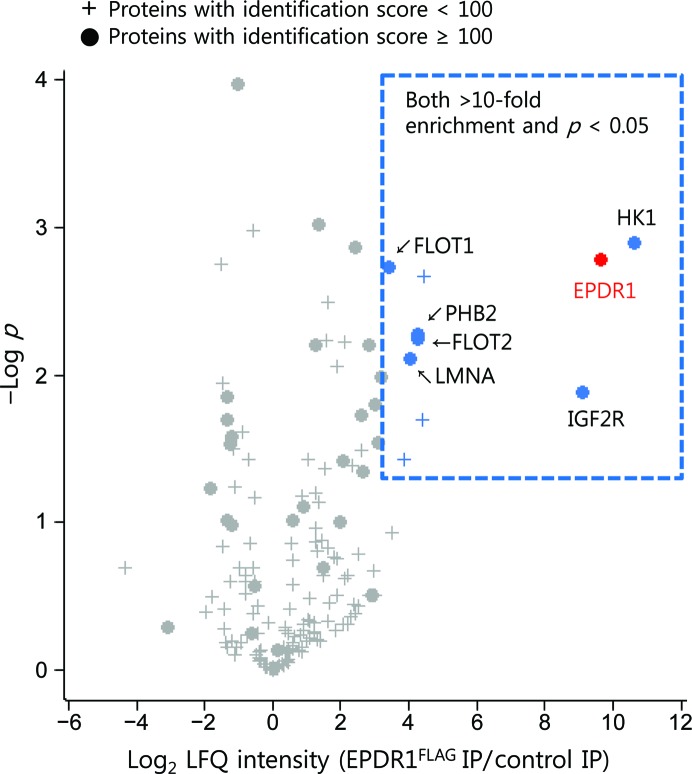
Volcano plot for the proteins quantified by EPDR1^FLAG^ immunoprecipitation–mass spectrometry (IP-MS). LFQ intensity values for EPDR1 (depicted as a red circle) and six other proteins (blue circles) increased in anti-FLAG IP-MS over control IP-MS (>10-fold, *p* < 0.05 after Student’s t-test). To calculate the statistical significance, LFQ intensity values for proteins that were not quantified in control IP-MS were replaced by random numbers drawn from a normal distribution (width, 0.3; down shift, 1.8) using *Perseus*. The protein-identification score determined by *MaxQuant* was applied to filter out proteins that are likely to be false-positive interactors.

Since the structure of EPDR1 resembles the fold of bacterial lipoprotein carrier proteins (LolA/LolB), with the hydrophobic binding pocket designed for binding lipid-anchored proteins, an interactome analysis of EPDR1 was performed to determine whether any known lipid-modified proteins bind to EPDR1. For this analysis, FLAG-tagged EPDR1 transiently expressed in U-87MG cells was used. U-87MG cells were selected for exogenous EPDR1 expression because the expression levels of both endogenous (http://www.proteinatlas.org; Uhlen *et al.*, 2010 ▸) and exogenous (Supplementary Fig. S9) EPDR1 in these cells seemed suitable for mass spectrometry. As the majority of exogenously expressed EPDR1 (EPDR1^FLAG^) was found to be secreted into cell-culture medium after expression (Supplementary Fig. S9), EPDR1^FLAG^ in the culture medium was pre-bound to anti-FLAG antibody-conjugated beads. As the initial aim was to identify the EPDR1 interactors in the cell, whole cell lysates prepared from U-87MG cells that were not subjected to transient EPDR1^FLAG^ expression were used to pull down the EPDR1 interactors because minimizing the total EPDR1 protein in the lysate that may compete with bead-bound EPDR1^FLAG^ will enhance our chance of capturing EPDR1 interactors *in extracto*. Immunoprecipitation–mass spectrometry (IP-MS) analysis and statistical filtering identified six proteins as candidate EPDR1 interactors (Fig. 6 ▸). All of these proteins displayed an at least tenfold greater abundance in EPDR1^FLAG^ IP-MS than in control IP-MS, with *p* values of less than 0.05.

Among the candidate EPDR1 interactors, IGF2R was the most prominent EPDR1 binder based on the MS/MS values observed in EPDR1^FLAG^ IP-MS (Supplementary Table S1). IGF2R (insulin-like growth factor 2 receptor, also called cation-independent MPR) is a 2491-residue transmembrane receptor that transports protein cargos earmarked with M6P into lysosomes (Brown *et al.*, 2009 ▸). As human EPDR1 has been reported to be modified by M6P and to localize into lysosomes (Sleat *et al.*, 1996 ▸, 2005 ▸, 2006 ▸; Kollmann *et al.*, 2005 ▸; Tribl *et al.*, 2005 ▸; Lübke *et al.*, 2009 ▸), the identification of IGF2R confirms that the expressed EPDR1 is M6P-modified and that IGF2R is the main M6P receptor utilized by M6P-modified EPDR1 for its proper lysosomal localization. The identification of IGF2R also indicates that the interactome analysis is valid.

Other interesting EPDR1 interactors on the list with specific subcellular locations in endosomal or plasma membranes were FLOT1 (flotillin-1) and FLOT2 (flotillin-2). The two proteins share 48% sequence identity and co-assemble to form multimeric protein complexes on the membranes of the late endosomes and lysosomes (Stuermer *et al.*, 2001 ▸; Langhorst *et al.*, 2005 ▸; Babuke *et al.*, 2009 ▸; Riento *et al.*, 2009 ▸). Flotillin proteins are reported to be involved in the retraction of plasma membrane vesicles and the endocytosis of certain proteins (Babuke *et al.*, 2009 ▸; Aït-Slimane *et al.*, 2009 ▸; Cremona *et al.*, 2011 ▸). Moreover, it is known that FLOT1 undergoes palmitoylation (Morrow *et al.*, 2002 ▸) and FLOT2 undergoes both palmitoylation and myristoylation (Neumann-Giesen *et al.*, 2004 ▸). Although it remains to be seen whether EPDR1 interacts with flotillin *via* the lipid anchors, judging from their strong interactions with EPDR1 it can be assumed that flotillin proteins may be a key component in IGF2R-mediated lysosomal translocation of EPDR1 or may be related to EPDR1 function.

## Supplementary Material

PDB reference: frog EPDR1, 6jl9

PDB reference: mouse EPDR1, 6jla

PDB reference: human EPDR1, 6jld

Supplementary Table and Figures. DOI: 10.1107/S2052252519007668/tj5023sup1.pdf

## Figures and Tables

**Table 1:** Data-collection and refinement statistics for EPDR1 Values in parentheses are for the highest resolution shell.

EPDR1	Frog (Xe phasing)	Frog (native)	Mouse (native)	Human (native)
Data collection				
Date	25/7/2018	14/6/2018	14/6/2018	22/12/2017
Diffraction source	7A, PLS	5C, PLS	5C, PLS	7A, PLS
Space group	*P*6_5_22	*P*6_5_22	*P*2_1_	*P*4_3_
Detector	ADSC Q270	ADSC Q315	ADSC Q315	ADSC Q270
Wavelength (Å)	1.54001	1.00930	1.00650	0.97935
Oscillation (°)	1	1	1	1
No. of frames	360	180	360	180
*a*, *b*, *c* (Å)	61.46, 61.45, 233.84	61.21, 61.21, 236.20	57.00, 59.67, 137.34	55.86, 55.86, 273.77
α, β, γ (°)	90, 90, 120	90, 90, 120	90, 101.29, 90	90, 90, 90
Resolution (Å)	50–2.90 (2.95–2.90)	50–2.00 (2.03–2.00)	50–2.40 (2.44–2.40)	50–2.00 (2.03–2.00)
*R*_merge_ (%)	9.7 (58.2)	8.3 (103.6)	8.3 (42.2)	8.1 (106.3)
*R*_p.i.m._ (%)	2.1 (12.5)	2.0 (23.0)	3.4 (16.5)	3.2 (41.8)
CC_1/2_	(0.976)	(0.911)	(0.988)	(0.686)
〈*I*/σ(*I*)〉	71.0 (10.5)	70.5 (4.5)	44.4 (6.9)	37.7 (2.3)
Completeness (%)	99.9 (100.0)	99.7 (100.0)	99.9 (100.0)	99.8 (100.0)
Multiplicity	21.9 (22.5)	19.8 (20.9)	7.2 (7.5)	7.5 (7.4)
Unique reflections	6390	18642	35643	56296
Overall *B* factor from Wilson plot (Å^2^)	63.6	41.0	41.1	45.2
Initial phasing FOM (*PHENIX*)	0.36	—	—	—
Refinement				
Resolution (Å)	—	2.0	2.4	2.0
NCS molecules in asymmetric unit	1	1	4	4
*R*_work_/*R*_free_ (%)	—	19.4/27.8	18.5/24.0	19.9/23.5
No. of atoms				
Protein	—	1565	6092	5981
Ligand/ion	4 Xe	1 Ca	6 NAG, 1 FUC	
Water	—	57	109	166
*B* factors (Å^2^)				
Protein (main chain/side chain)	—	51.6/59.7	49.7/58.1	51.9/58.2
Water	—	54.9	45.0	54.8
R.m.s. deviations				
Bond lengths (Å)	—	0.014	0.016	0.018
Bond angles (°)	—	1.74	1.84	1.98
