# Supplementary material for: Structures of three ependymin-related proteins suggest their function as a hydrophobic molecule binder
Source: IUCrJ. 2019 Jun 20;6(Pt 4):729–39. doi: 10.1107/S2052252519007668 (PMC6608618; doi:10.1107/S2052252519007668)
Supplement: Supplementary file 1 [file m-06-00729-sup1.pdf]

IUCrJ

Volume 6 (2019)

Supporting information for article:

Structures of three ependymin-related proteins suggest their function as a hydrophobic molecule binder

Jeong Kuk Park, Keon Young Kim, Yeo Won Sim, Yong-In Kim, Jin Kyun Kim, Cheol Lee, Jeongran Han, Chae Un Kim, J. Eugene Lee and SangYoun Park

Table S1 Summary of proteins associated with EPDR1^{FLAG}

Gene names	Fold-change	*p*-value[a]	LFQ intensity[b]		MS/MS count[c]		Subcellular location[d]
			Control	EPDR1^{FLAG}	Control	EPDR1^{FLAG}	
EPDR1	785	0.002	0	3.33×10^{9}	0	159	Extracellular region or secreted
IGF2R	540	0.013	5.57×10^{6}	3.26×10^{9}	0	469	Lysosome membrane
HK1	1600	0.001	1.42×10^{6}	2.21×10^{9}	0	285	Mitochondrion
FLOT2	19.5	0.006	1.77×10^{8}	3.45×10^{9}	1	335	Endosome, Plasma membrane
PHB2	19.3	0.005	1.64×10^{7}	3.10×10^{8}	0	63	Nucleus
LMNA	16.6	0.008	2.27×10^{7}	3.69×10^{8}	0	118	Nucleus
FLOT1	10.6	0.002	2.72×10^{8}	2.89×10^{9}	8	380	Endosome, Plasma membrane

[a]These values were determined by Student's t-test. See also Fig. 6.

[b]LFQ intensity values are average of biological duplicate.

[c]MS/MS values are sum of biological duplicate.

[d]From Uniprot database

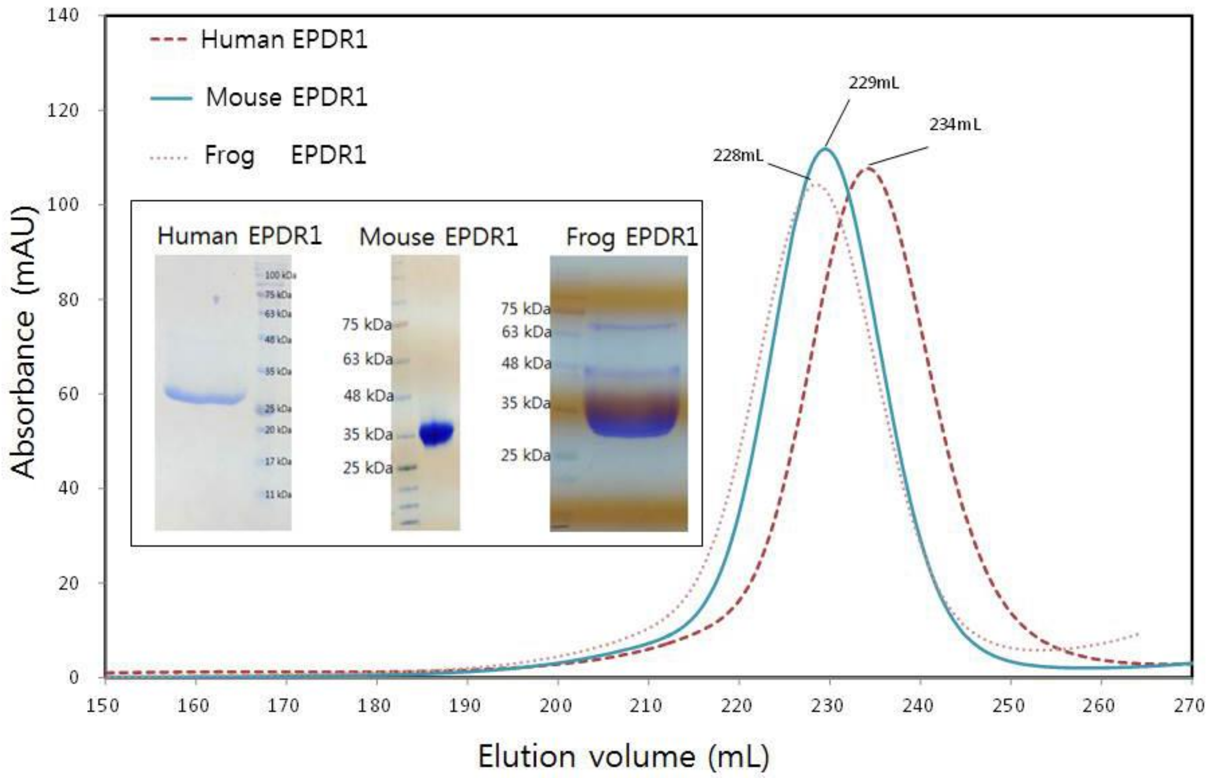

Figure S1 Purifications of human, mouse, and frog EPDR1s using size-exclusion gel filtration chromatography. The FPLC-chromatograms at the last stage of purifications are shown for the human, mouse and frog EPDR1s. The SDS-PAGE results of the concentrated proteins are also shown (inset).

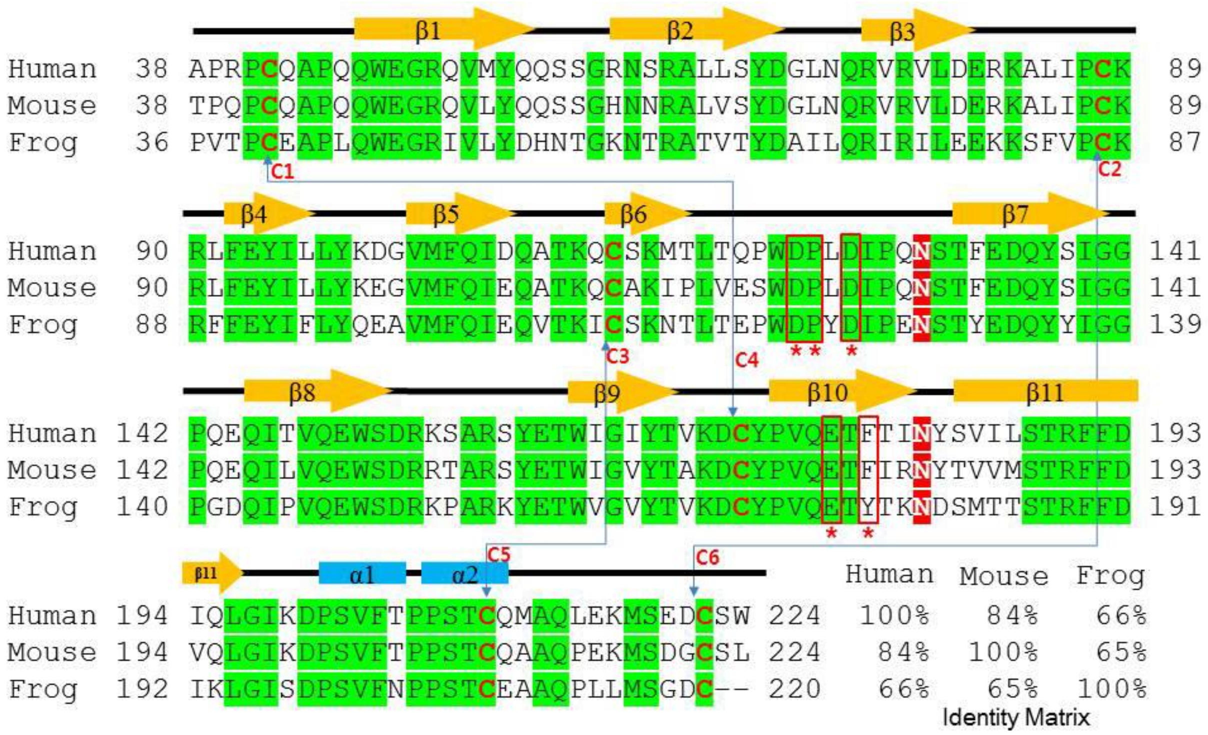

Figure S2 Structure-based sequence alignment of human, mouse, and frog EPDR1s. Sequences of human, mouse, and frog EPDR1s are aligned based on the determined structures with secondary structural elements shown above the alignment. Identically conserved residues are shown with green background, and asparagine residues found glycosylated are shown with red background. All six cysteine residues (C1-C6) participate in forming three disulfide bonds. Sequence identity matrix relating the three sequences are also shown.

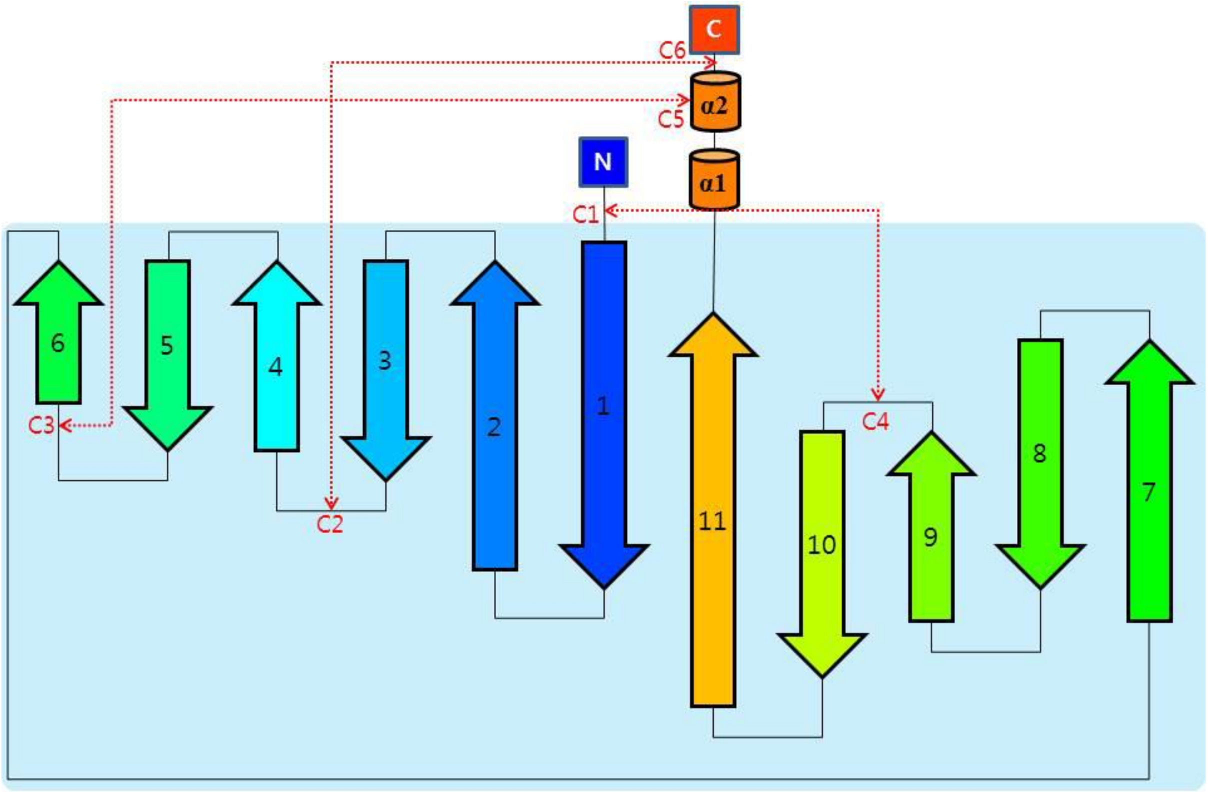

Figure S3 Topological diagram of the EPDR1 fold. The topological diagram of the monomeric subunit of human EPDR1 illustrates that the 11 β-strands are arranged in an anti-parallel fashion. The positions of the cysteines (C1-C6) and their mediating disulfide bonds are shown with red arrows. The frog EPDR1 residues (Asp121, Pro122, Asp124, Glu175 and Tyr177) which are involved in stabilizing a bound metal (modeled as Ca^{2+}) are indicated using red asterisks, and the aligned residues boxed in red.

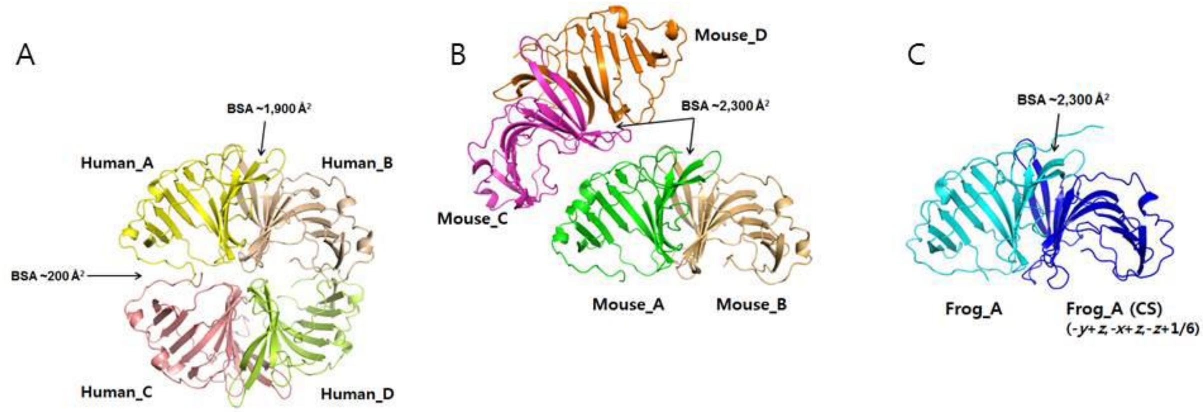

Figure S4 The oligomeric interactions in the three EPDR1s (human (A), mouse (B), and frog (C)). (A) Human EPDR1 crystal contained four EPDR1 molecules in the crystal asymmetric unit. Buried surface areas (BSA) of ~1,900 Å^2 (per subunit) were found between the two dimers. The smaller interface which buries ~200 Å^2 (per subunit) appears to be only a lattice contact. (B) Mouse EPDR1 crystal also contained four EPDR1 molecules in the crystal asymmetric unit. The same dimeric interfaces with BSA of ~2,300 Å^2 (per subunit) relate the monomeric units in the two dimers. (C) Frog EPDR1 crystal only contained one EPDR1 molecule in the crystal asymmetric unit. However, the same dimeric interface of the human and mouse EPDR1s mediates the dimeric interaction between the two crystallographic symmetry mates.

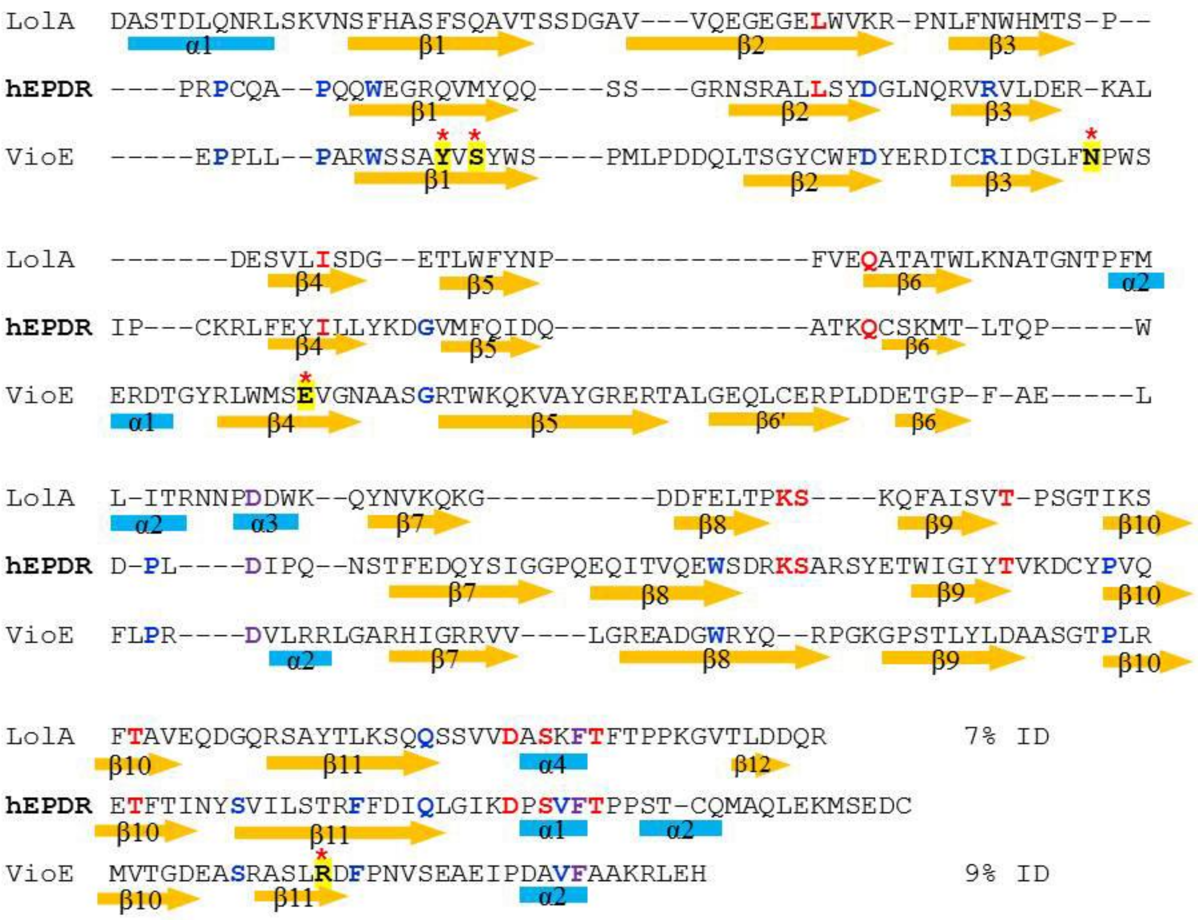

Figure S5 Structure-based sequence alignment of human EPDR1, and bacterial LolA and VioE. Sequences of human EPDR1 (hEPDR, *this study*), LolA (PDB code 4KI3), and VioE (PDB code 3BMZ) are aligned based on the structures with secondary structural elements shown above the sequence. Percentage sequence identities (ID) of human EPDR1 against LolA (7%) or VioE (9%) are also indicated. Residues necessary for VioE enzyme activity (Tyr17, Ser19, Asn51, Glu66, and Arg172), which are not conserved in EPDR1s are indicated in yellow background with red asterisks. The structural locations of these residues are indicated in SFig. 6.

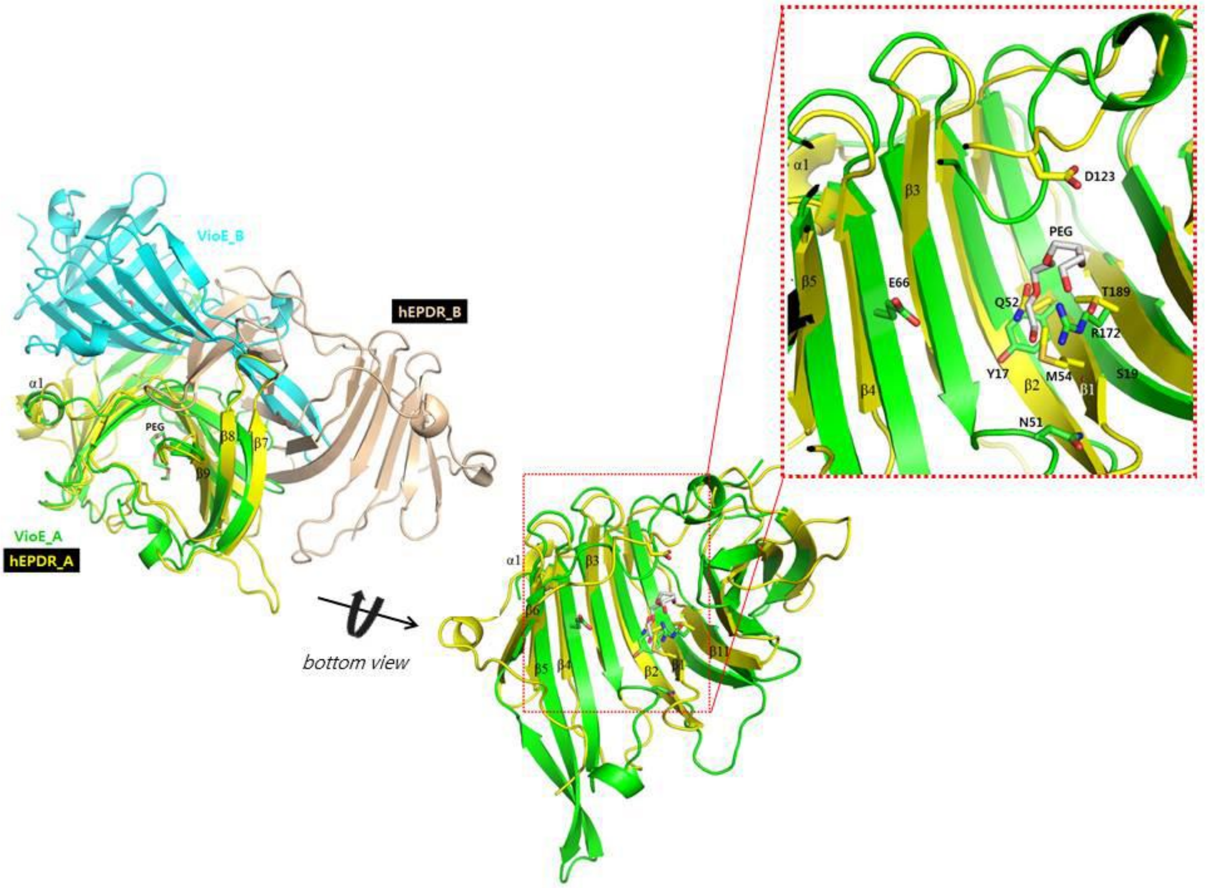

Figure S6 Structural superposition of human EPDR1 (hEPDR) dimer and VioE (PDB code 3BMZ) dimer, and the view near the hydrophobic pocket. Despite the low level of sequence similarity, the topologies of the two stacked β-sheet layered structures made from the 11 anti-parallel β-strands are closely related in hEPDR1 and VioE. However, the dimeric interfaces are not conserved between the two proteins. A PEG molecule (in white) placed in the hydrophobic pocket was observed in VioE. Although several residues near the bound PEG have been assigned to be critical for VioE's enzymatic activity (Tyr17, Ser19, Asn51, Glu66, and Arg172), none of these residues are conserved in hEPDR1. Critical VioE residues as well as the aligned residues of hEPDR1 (Gln52, Met54, and Thr189 are shown. The location of hEPDR1 Asp123 which may interact with Ca^{2+} as in the case of frog EPDR1 is also indicated.

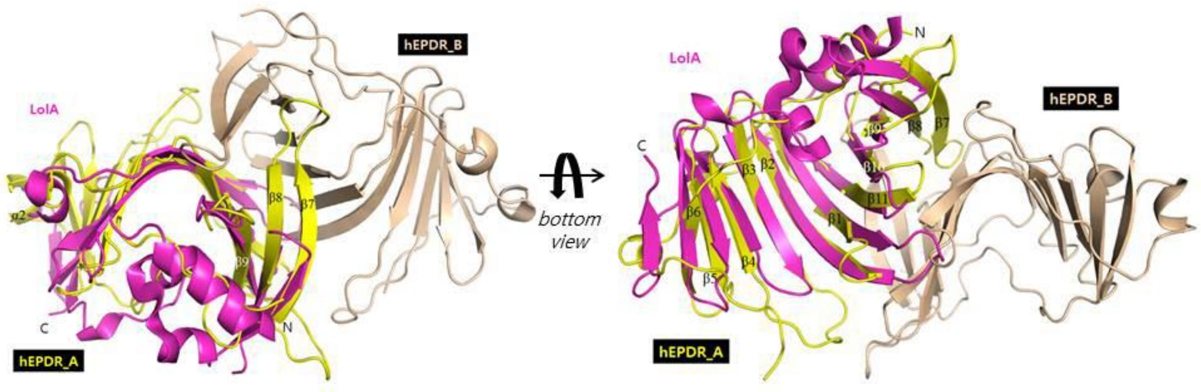

Figure S7 Structural superposition of human EPDR1 (hEPDR) dimer and LolA (PDB code 4KI3) monomer. Despite the low level of sequence similarity, the topologies of the two stacked β-sheet layered structures made from the 11 anti-parallel β-strands are closely related in hEPDR and a lipoprotein carrier protein LolA. Unlike VioE, LolA does not undergo dimeric interaction in the crystal.

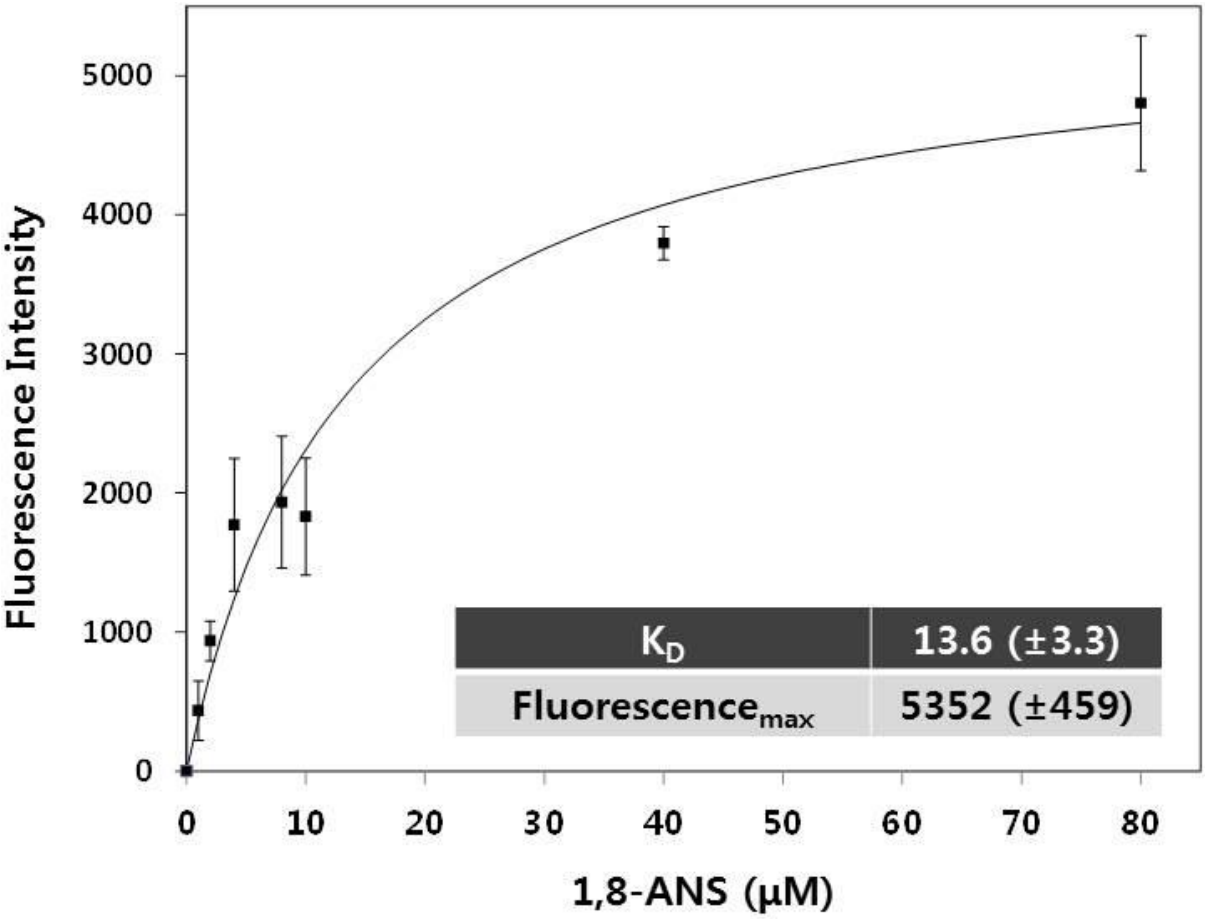

Figure S8 Binding of a fluorescent probe 1,8-ANS to human EPDR1. In order to assess the binding of fatty acids to human EPDR1, 1,8-ANS was used as a fluorescent probe for the displacement experiment. Curve fitting analysis suggests that 1,8-ANS binds to human EPDR1 with K_D ~14 μM.

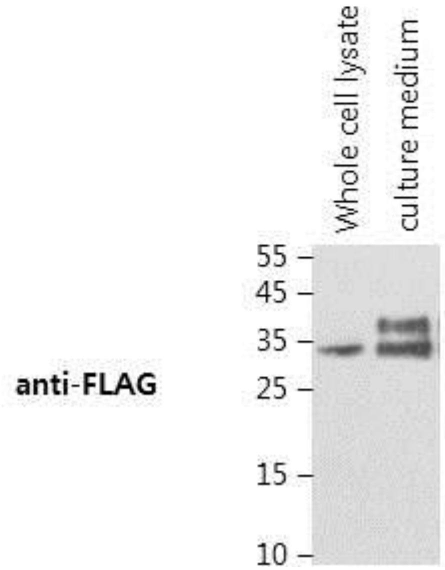

Figure S9 EPDR1^{FLAG} expressions in U-87MG cells. Whole cell lysate and culture medium from U-87MG cells transiently expressing EPDR1^{FLAG} were subjected to immunoblot analysis with anti-FLAG antibodies. The culture medium containing the secreted EPDR1^{FLAG} was concentrated using 10-kDa molecular weight cut-off filters. The upper band in the culture medium lane is likely a differently glycosylated form of EPDR1^{FLAG}.
